# Epistaxis as a Common Presenting Symptom of Glanzmann's Thrombasthenia, a Rare Qualitative Platelet Disorder: Illustrative Case Examples

**DOI:** 10.1155/2017/8796425

**Published:** 2017-02-19

**Authors:** Michael Recht, Meera Chitlur, Derek Lam, Syana Sarnaik, Madhvi Rajpurkar, David L. Cooper, Sriya Gunawardena

**Affiliations:** ^1^The Hemophilia Center at Oregon Health & Science University, Portland, OR, USA; ^2^Carman and Ann Adams Department of Pediatrics, Wayne State University and Children's Hospital of Michigan, Detroit, MI, USA; ^3^Children's Hospital of Michigan, Detroit, MI, USA; ^4^Clinical Development, Medical and Regulatory Affairs, Novo Nordisk Inc., Plainsboro, NJ, USA

## Abstract

Children often present to emergency departments (EDs) with uncontrollable nose bleeding. Although usually due to benign etiologies, epistaxis may be the presenting symptom of an inherited bleeding disorder. Whereas most bleeding disorders are detected through standard hematologic assessments, diagnosing rare platelet function disorders may be challenging. Here we present two case reports and review diagnostic and management challenges of platelet function disorders with a focus on Glanzmann's thrombasthenia (GT). Patient 1 was a 4-year-old boy with uncontrolled epistaxis. His medical history included frequent and easy bruising. Previous laboratory evaluation revealed only mild microcytic anemia. An otolaryngologist stopped the bleeding, and referral to a pediatric hematologist led to the definitive diagnosis of GT. Patient 2 was a 2.5-year-old girl with severe epistaxis and a history of milder recurrent epistaxis. She had a bruise on her abdomen with a palpable hematoma and many scattered petechiae. Previous assessments revealed no demonstrable hemostatic anomalies. Platelet aggregation studies were performed following referral to a pediatric hematologist, leading to the diagnosis of GT. As evidenced by these cases, the ED physician may often be the first to evaluate severe or recurrent epistaxis and should recognize indications for coagulation testing and hematology consultation/referral for advanced hematologic assessments.

## 1. Introduction

Recurring epistaxis is a common occurrence in children, estimated to affect up to 9% of the pediatric population [1]. In most cases, bleeding can be controlled by applying firm, constant pressure over the lower part of the nose for 20 minutes [2]. However, in cases of severe or prolonged bleeding, children may present to the emergency department (ED) and require therapeutic intervention. Epistaxis is a frequent symptom of children presenting to the ED, and it is estimated to account for approximately 4 in 1000 ED visits among children in the United States [3].

The nose is a vulnerable site for bleeding as the nasal mucosa is richly supplied with vessels that form plexiform networks. Most episodes of epistaxis in children are caused by mucosal dryness and exposure of an anterior septal blood vessel, trauma (including nose picking), or retained foreign bodies. Less commonly, nosebleeds can also occur due to local disturbances such as infections of the upper respiratory tract or inflammation (allergic rhinitis). The presence of an underlying bleeding disorder is an important consideration when evaluating children with severe or recurrent epistaxis, as an estimated one-third of patients with recurrent epistaxis have an underlying hemostatic disorder [4]. Because emergency providers may often be the first line of evaluation of a patient with recurrent epistaxis, it is important that they be aware of potential underlying hemostatic disorders. Furthermore, emergency providers should recognize the clinical signs indicating when hematologic laboratory assessments or specialist consultation may be necessary.

Types of bleeding disorders that may present with epistaxis include coagulation factor deficiencies, Von Willebrand disease, and several rare inherited platelet function disorders associated with defects in specific aspects of platelet function (Table 1). Platelet function disorders vary in severity, with some of the most severe including Glanzmann's thrombasthenia (GT) and Bernard-Soulier syndrome; however, other conditions which are generally milder may also occasionally produce severe bleeding symptoms. Epistaxis and other mucocutaneous types of bleeding are more commonly associated with Von Willebrand disease and platelet function disorders than with coagulation factor deficiencies and may often comprise the presenting symptoms leading to a bleeding disorder diagnosis.

Among the severe inherited platelet function disorders, GT is the most common [5], usually manifesting in infancy as severe or prolonged bleeding [6]. This autosomal recessive bleeding disorder is caused by a deficiency of or abnormality in the platelet membrane glycoproteins IIb or IIIa (GPIIb/IIIa). In contrast to unaffected individuals, in which activated GPIIb/IIIa mediates cross-linking of platelets into aggregates (clumps), individuals with GT exhibit impaired platelet-platelet interactions and insufficient clot formation [7]. Bleeding episodes in individuals with GT are usually mucocutaneous, and symptoms most frequently include purpura, epistaxis, gingival hemorrhage, and heavy menstrual bleeding [7–10].

Epistaxis is a commonly reported symptom in children with GT; however, because of the frequent occurrence of mild epistaxis in children with normal hemostatic function, screening for bleeding disorders in children with controllable episodes may often be overlooked [8]. Understanding the symptoms and laboratory assessments leading to a diagnosis of GT is therefore critical to identifying and properly managing this rare disorder. Here, we present two case reports of children with mild and severe GT who presented to the ED with epistaxis and review the referral pathways, differential diagnoses, and hemostatic assessments performed. These example cases may be a useful guide for emergency providers when considering whether children presenting with epistaxis may require hematologic assessments or specialist referral.

## 2. Patient Cases

### 2.1. Case  1

A 4-year-old boy presented to the ED experiencing a third episode of severe epistaxis in that day; this episode had been continuing for approximately one hour. He had also experienced another spontaneous episode approximately three weeks earlier, which like the first two on this presentation resolved at home with application of pressure. He had no history of a runny nose, cough, fever, or excessive picking at the nose.

The patient had been born after a full-term pregnancy without complications except for a nuchal cord at the time of delivery. Petechiae seen around the face and neck at the time of delivery were ascribed to the nuchal cord. He had experienced gastrointestinal reflux as an infant, and during severe episodes, petechiae of the face, neck, and trunk were noted. Chest bruises were noted at his 12-month well-child exam in a pattern consistent with the pressure applied by his car seat, leading to a laboratory workup to screen for coagulation disorders. The laboratory evaluation revealed mild microcytic anemia, a normal platelet count, and normal prothrombin time (PT) and activated partial thromboplastin time (aPTT). The patient was started on iron supplementation for anemia, which was ascribed to poor iron levels in breastmilk, as his mother was noted to have discontinued taking prenatal vitamins. Frequent episodes of epistaxis had begun around two years of age, in which bleeding would last for up to two hours, came from either or both nostrils, and occurred as often as weekly but never required medical or surgical intervention.

Upon presentation to the ED, the patient was noted to be anxious and slightly pale. His physical exam showed active bleeding from both nostrils and scattered petechiae around the orbits, chest, and extremities. Prolonged external compression of the nares with administration of a vasoconstrictive spray was insufficient to control the patient's epistaxis, and therefore, anterior and posterior nasal packing was performed. Through these measures the emergency physician was able to control the epistaxis for only a few minutes before bleeding resumed, and he requested the assistance of an otolaryngologist. Laboratory studies were also obtained and showed no anomalies except for microcytic anemia, with a normal PT/PTT and platelet count. The otolaryngologist was able to achieve hemostasis by cautery with silver nitrate and subsequently applied petroleum jelly to stabilize the clot and to prevent further bleeding. He referred the patient to a pediatric hematologist for further evaluation, due to a high suspicion of a bleeding disorder with the combination of recurrent epistaxis and petechiae.

The pediatric hematologist obtained further workup. The patient's CBC revealed a hemoglobin level of 9.8 g/dL, a mean corpuscular volume of 69 fL, and a platelet count of 435,000/*μ*L. Ferritin levels were low (8 ng/mL), consistent with iron-deficiency. Von Willebrand studies were normal (Von Willebrand factor [VWF] antigen, 112 IU/dL; ristocetin cofactor, 120 IU/dL; factor VIII [FVIII] activity, 98 IU/mL). The patient had a normal PT and PTT and fibrinogen level. Platelet Function Analyzer 100 (PFA-100) closure times were prolonged with both collagen/epinephrine and collagen/adenosine diphosphate (ADP). Platelet aggregation testing revealed abnormal platelet aggregation with a lack of response to all agonists (ADP, epinephrine, and collagen) except ristocetin (present but incomplete response). Follow-up platelet immunophenotyping using flow cytometry demonstrated decreased expression of CD41 (a marker of platelet membrane GPIIb) and normal expression of CD61 (a marker of GPIIIa) and CD42b (a marker of GPIb), suggesting a specific deficiency in GPIIb and confirming the diagnosis of GT.

### 2.2. Case  2

A 2.5-year-old girl presented to the ED with severe epistaxis that had been continuing for approximately one hour. The patient's history indicated that she had experienced no umbilical cord bleeding at birth, but she was noted to bruise easily throughout infancy and childhood. She had begun experiencing recurrent epistaxis at 14 months of age and had approximately two to three bleeding episodes each month, typically lasting 15 to 20 minutes, with no seasonal variation. She had never previously visited a physician or ED to control her nosebleeds. Prior episodes of epistaxis were easily controlled with applied pressure at home and without the need for therapeutic intervention by a health care professional.

The patient's parents had consulted with their child's pediatrician at her 18-month well-child visit and were advised to monitor her bleeding symptoms. Because of the patient's recurring epistaxis and easy bruising, the pediatrician ordered laboratory testing at her 2-year well-child exam. Laboratory studies demonstrated a normal CBC, platelet count, and PT/PTT.

On presentation to the ED, the patient appeared to be a well-nourished, playful toddler without any distress. She was afebrile, and her other vital signs were normal. She had dried blood around her left naris with no evidence of active bleeding from either nostril. She exhibited a large (2 × 2-cm) bruise on her lower abdomen with a palpable hematoma underneath with several petechiae in various stages of resolution on the trunk, extremities, and face. The rest of her physical exam was normal.

Because of the patient's recurrent epistaxis, bruising, and petechiae, the ED provider, an otolaryngologist, and a pediatric hematologist were consulted. Bleeding was controlled using vasoconstrictive spray and nasal packing. Laboratory testing was also performed as per the hematologist's recommendation. The patient's hematologic assessment revealed similar laboratory findings as were noted for Patient 1; her white blood cell and platelet counts were normal, although her hemoglobin was low. Her ferritin level was also low. Coagulation studies showed a normal PT and PTT and no evidence of Von Willebrand disease (VWF antigen, 107 IU/dL; ristocetin cofactor, 116 IU/dL; FVIII activity, 97 IU/mL). Her platelet aggregation studies showed greatly reduced responses with all agonists except for agglutination to ristocetin, indicative of GT. A diagnosis of GT was confirmed after immunophenotyping analysis using flow cytometry at the follow-up hematology visit, which demonstrated reduced expression of CD41 (GPIIb) and CD61 (GPIIIa).

## 3. Discussion

Although epistaxis is a common occurrence in children and often improves with age, severe or recurrent epistaxis can be the presenting symptom of children with underlying bleeding disorders [1, 4]. Because nonhematologist physicians and allied health professionals are less likely to encounter individuals with platelet function disorders as opposed to other bleeding disorders, such as Von Willebrand disease and coagulation factor deficiencies, the identification of patients who may benefit from advanced hematologic assessments can be challenging.

GT is the most common of the severe, inherited platelet function disorders. Therefore, reviewing the diagnostic and referral pathways of children with GT presenting to the ED may serve as an example of the considerations most relevant to hospital-based physicians. The incidence of GT is estimated to be approximately 1 in 1 million; however, the disorder is found more commonly in individuals from ethnic groups in which consanguinity is common [11]. Like other platelet function disorders, GT commonly involves mucocutaneous bleeding, which manifests in children as epistaxis, bruising, and petechiae [11]. Children with GT experience prolonged nosebleeds that may be difficult to control with typical methods. Accurate and timely diagnosis is important for enabling physicians to properly manage the medical needs of individuals with GT, including ensuring regular dental care to prevent gingival bleeding, preparing for potentially heavy menstrual bleeding in young females, monitoring for iron-deficiency anemia, and considering hemostatic requirements for any necessary surgeries.

### 3.1. Initial Management and Specialist Referrals

Epistaxis episodes in children are usually benign. However, some patients with severe or recurrent episodes may present with evidence of volume depletion or airway compromise prompting emergent management. Initial approaches towards managing epistaxis in a hemodynamically stable patient typically include compression of the nostrils and application of topical vasoconstrictors [12, 13]. Patients should be instructed to tilt their head forward to prevent airway obstruction and swallowing of blood. If bleeding is not controlled after these initial steps, physicians should attempt to locate the source of bleeding and also try to determine the etiology of bleeding (i.e., local versus systemic causes).

Subsequent steps to control anterior nasal bleeding most often include vasoconstriction, in which cotton pledgets soaked in vasoconstrictor and local anesthetic are placed in the anterior nasal cavity and direct pressure is applied to both sides of the nose for several minutes. If bleeding continues, more aggressive approaches may include chemical cautery using silver nitrate, electrocautery, hemostatic packing with absorbable gelatin foam or oxidized cellulose, or treatment with a fibrin glue. Posterior bleeding is less common and often more severe than anterior bleeding and is usually treated by an otolaryngologist; management approaches typically involve posterior packing or alternative packing strategies using various balloon systems. The most persistent cases of anterior or posterior bleeding may require arterial ligation or embolization.

In addition to managing acute bleeding symptoms, emergency health care providers should be aware of the clinical indications that a bleeding disorder may be present. Coagulation disorders should be considered in children with a history of recurrent, prolonged, severe, or refractory bleeding and for individuals with a history of easy bruising, other severe bleeding, or gingival bleeding [12, 13]. The presence of petechiae may be a particularly visible indication of additional bleeding symptoms. Previous inpatient admissions or emergency care for bleeding symptoms, the presence of iron-deficiency anemia, and a family history of excessive bleeding may also be useful in identifying those at an increased risk for bleeding disorders. Suspected cases of platelet disorders should be referred to a hematologist even if initial hematologic assessments, including blood counts and PT/PTT, appear normal.

### 3.2. Hematologic Assessments

In cases of severe epistaxis presenting to the ED, preliminary laboratory studies, including a CBC and PT/aPTT, are typically requested by the emergency physician or by an otolaryngologist. PT and aPTT levels and platelet counts and morphology are normal in individuals with GT, although recurrent blood loss frequently results in low hemoglobin or anemia. The closure time (CT), measured by a PFA-100, may also be used as a screening test in some institutions, although this system lacks specificity for GT and platelet function disorders [14]. PFA-100 CT measures the time required for platelets from the patient's blood sample to form a platelet plug in response to collagen and platelet agonists. An absent or delayed closure time may be a preliminary indication of a platelet function disorder or Von Willebrand disease. This system should therefore detect abnormalities in individuals with GT or other severe platelet function disorders; however, additional testing is necessary to confirm a diagnosis [14]. It is important to note that the PFA-100 may not be abnormal in those with mild Von Willebrand disease or mild qualitative platelet dysfunction.

Following initial laboratory studies, patients with a suspected platelet function disorder are typically referred to a hematologist for additional testing. Platelet aggregometry is the current gold standard for the diagnosis of platelet function disorders. This tool measures the change in light transmission through a sample of platelet-rich plasma when aggregation agonists are added or measures the increase in resistance to the passage of electric current through a whole blood sample with the addition of an agonist [15]. GT can be distinguished from other platelet function disorders by a lack of platelet aggregation in response to all agonists and a normal response to ristocetin; unlike agonists which induce platelet aggregation (requiring functional GPIIb/IIIa), ristocetin induces platelet agglutination through a facilitation of VWF binding to glycoprotein Ib and therefore serves as a measure of VWF activity. An important consideration in performing platelet function studies is the patient's use of medications that may alter platelet function (but would not have accounted for the pattern of laboratory results seen in patients with GT), including, but not limited to, COX-1 and COX-2 inhibitors, beta-lactam antibiotics, and selective serotonin reuptake inhibitors [16].

Flow cytometry may also be helpful in diagnosing GT, by assessing surface levels of CD41 (indicating GPIIb) and CD61 (indicating GPIIIa). As GPIIb/IIIa forms a complex on the platelet surface for binding to fibrinogen and causes aggregation of platelets, low or absent GPIIb or GPIIIa is characteristic of GT. Flow cytometry may also be useful for determining the carrier status of family members [17].

**Figure 1:**
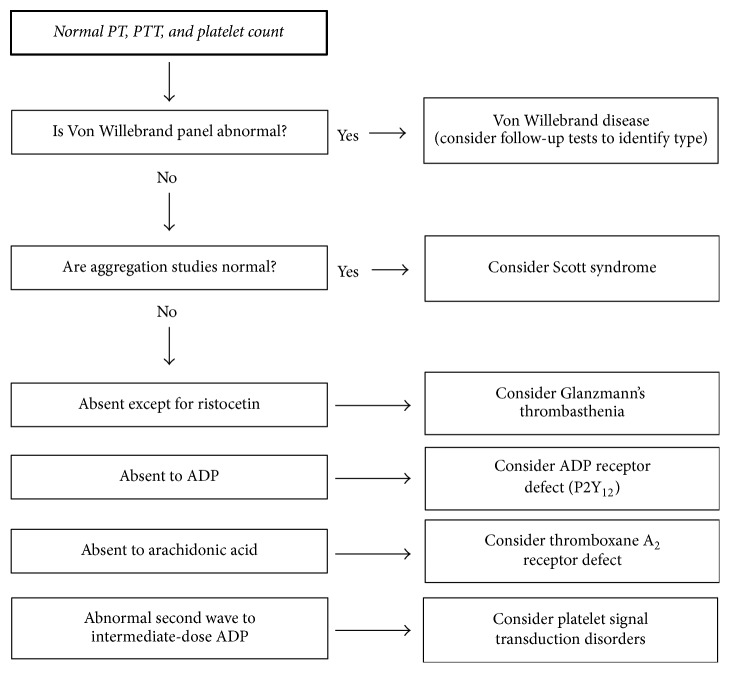
Algorithm for the evaluation and differential diagnosis of bleeding disorders in children presenting with epistaxis and a normal PT, PTT, and platelet count. ADP, adenosine diphosphate; PT, prothrombin time; PTT, partial thromboplastin time.

Key to the diagnosis of GT and other platelet disorders is the exclusion of other coagulation disorders that may cause similar bleeding phenotypes. A diagnostic algorithm describing the series of laboratory assessments underlying the differential diagnosis of bleeding disorders in children presenting with epistaxis and a normal PT, PTT, and platelet count is shown in Figure 1. This stepwise series of laboratory tests is critical for obtaining an accurate diagnosis of rare platelet function disorders.

## 4. Conclusions

A substantial number of children in the United States experience recurrent epistaxis, and emergency physicians often encounter those experiencing severe bleeding. The emergency physician may often be the first physician to evaluate a patient's epistaxis and therefore should recognize the clinical indications for hematologic testing, consultation, and referral. An early and accurate diagnosis of GT or other rare bleeding disorders is important for ensuring proper disease management and long-term care.

## Figures and Tables

**Table 1:** Types of congenital platelet function disorders.

Type of platelet function defect	Diagnoses	Protein or functional deficiency
Cytoskeletal assembly	Wiskott-Aldrich syndrome	Wiskott-Aldrich syndrome protein

Granule storage or release	Storage pool deficiencies	Granule platelet content

Platelet adhesion	Von Willebrand disease	VWF
	Bernard-Soulier syndrome	GPIb

Platelet aggregation	Glanzmann's thrombasthenia	GPIIb/IIIa
	Congenital hypofibrinogenemia	Fibrinogen

Platelet-agonist interactions	Agonist receptor deficiencies	ADP or thromboxane A_2_

Platelet coagulant-protein interactions	Scott syndrome	Platelet membrane phospholipid signaling

ADP, adenosine diphosphate; GP, glycoprotein; VWF, Von Willebrand factor.
